# Active surveillance of carbapenem-resistant Gram-negative healthcare-associated infections in a low-middle-income country city

**DOI:** 10.1016/j.bjid.2021.101540

**Published:** 2021-02-13

**Authors:** Viviane Maria de Carvalho Hessel Dias, Daniela Maria Waszak da Silva, Marion Burger, Alcides Augusto Souto de Oliveira, Patrícia de Jesus Capelo, Fabio Augusto da Rocha Specian, Marianna Cavina de Figueiredo, Felipe Francisco Tuon, Cristina Pellegrino Baena

**Affiliations:** a Pontifícia Universidade Católica do Paraná, Escola de Medicina, Curitiba, PR, Brazil; b Secretaria Municipal de Saúde de Curitiba, Curitiba, PR, Brazil; c Secretaria de Saúde do Estado do Paraná, Curitiba, Paraná, Brazil

**Keywords:** Healthcare-associated infections, Carbapenem resistance, Active surveillance, Low-middle-income countries

## Abstract

**Background:**

Carbapenem-resistance in healthcare-associated infections (HCAIs) is of great concern, and it is urgent to improve surveillance. We aimed to describe and analyze HCAIs trends on Gram-negative antimicrobial susceptibility in a city from a developing country, following the implementation of an active surveillance program.

**Methods:**

This is an aggregated study describing data from 24 hospitals with intensive care units, including a trend analysis by *Joinpoint* regression between January 2012 and December 2017.

**Results:**

There were 23,578 pathogens in 39,832 HCAIs, from which 16,225 were Gram-negatives (68.8%). Carbapenem susceptibility was lowest in *A. baumannii* (15.4–25.9%), *K. pneumoniae* (51.0–55.9%), and *P. aeruginosa* (64.9–84.1%) and highest in *E. coli* (96.5–99.2%). Only *K. pneumoniae* showed a significant *Joinpoint* at 95% confidence interval: −10.71% (−18.02; −2.75) from 2012 to 2014, *p* = 0.02, and 6.54% (−2.00; 15.83) from 2015 to 2017, *p* = 0.12, which was most influenced by urinary tract infections: −9.98% (−16.02; −3.48) from 2012 to 2014, *p* = 0.01, and 9.66% (−1.75; 22.39) from 2015 to 2017, *p* = 0.09.

**Conclusion:**

Although we found a significant change toward an improvement in carbapenem susceptibility in *K. pneumoniae*, resistance is high for most pathogens. These data should encourage health institutions to improve their prevention and control strategies.

## Introduction

The burden of healthcare-associated infections (HCAIs) in developing countries is of great concern, and there is an urgent need to improve surveillance and infection control practices.^1^

HCAIs caused by multidrug-resistant organisms (MDRO) are associated with high mortality rates and increased hospital length of stay and costs.^2^ Therefore, national, regional, and local continuous surveillance programs are important to assist health institutions on HCAIs and avoid the emergence of MDRO.^3^

The World Health Organization (WHO) has pointed out a priority list of antibiotic-resistant bacteria to guide research and development of new antibiotics. Carbapenem-resistant Gram-negative bacilli (*A. baumannii*, *P. aeruginosa*, and *Enterobacterales*) were listed as “number one priority” for implementation of active surveillance, prevention, and control actions in healthcare organizations.^4^ Interestingly, an European surveillance report from 2009 to 2012 showed that carbapenem-resistant *K. pneumoniae* occurrence varied from 0% to 60.5%, and carbapenem-resistant *P. aeruginosa* occurrence was >10% in 19 of 29 reporting countries.^5^

In Brazil, data on surveillance of MDRO related to HCAIs are limited, and incidence trends of HCAIs due to carbapenem-resistant *A. baumannii* has been described in a 12-month period in the city of Porto Alegre in southern Brazil.^6^ Based on that study, eight clonal groups were identified by molecular typing, and three of these were found in 12 public hospitals included in the report.

Data from the Brazilian SCOPE study (Surveillance and Control of Pathogens of Epidemiological Importance), showed that 58.5% of 2563 cases of bloodstream infections at 16 hospitals were caused by Gram-negative bacteria. Additionally, *Acinetobacter* spp. and *P. aeruginosa* isolates were resistant to carbapenem in 55.9% and 36.8% of cases, respectively.^7^

In Curitiba, another capital city located in south Brazil, the prevalence of MDRO in HCAIs was described for the first time in 2012 by the Municipal Health Department through the Working Group in HCAIs.^8^ In that study, analysis from 2011 showed that carbapenem-resistant *A. baumannii* and third-generation-cephalosporin-resistant *K. pneumoniae* were the most frequent MDRO in HCAIs and, carbapenem-resistant *Enterobacterales* prevalence in HCAIs varied from 2.1 to 8.0%.

Therefore, this study aimed to describe epidemiological trends and analyze possible changes in Gram-negative carbapenem susceptibility reported in HCAIs from January 2012 to December 2017, in the city of Curitiba, state of Paraná (PR), Brazil, following the implementation of an active surveillance program.

## Methods

### Study design

This is an aggregated data study that gathered information on HCAIs reports from the Municipal and State Health Departments of Curitiba, PR. This study was approved by the research ethics committee of Pontifícia Universidade Católica do Paraná (Certificate of Presentation for Ethical Appreciation, 87394318.0.0000.0020).

### Setting

This study was conducted in Curitiba, PR, a city of 434.89 km^2^ located in southern Brazil, with a population of 1,751,907 (2010), demographic density of 4027.04 inhabitants/km^2^ (2010), and per capita Gross Domestic Product of approximately U$ 8358.74 (2017), according to the Brazilian Institute of Geography and Statistics (Instituto Brasileiro de Geografia e Estatística).^9^

Data were abstracted from January 2012 through December 2017, following the implementation of an active surveillance program by the Curitiba Municipal Health Department in October 2010, through the Working Group in HCAIs. This municipal surveillance program consists of monthly notifications, by hospital infection control teams, of whole institution data on HCAIs etiological pathogens and sites such as bloodstream infection (BSI), urinary tract infection (UTI), respiratory tract infection (RTI), and surgical infection (SSI). All participating hospitals were instructed to notify the microorganisms only once per infection episode. The data was validated monthly by the team responsible for this monitoring at the municipal health department. No specific data were requested related to negative or unsolicited cultures. In addition to Municipal Health Department notification, institutions were also to report data on admissions and rate of HCAIs in a web-based platform monitored by the Paraná State Health Department.

As part of the surveillance program, to ensure adequate data collection, meetings were held with all hospitals involved. Time framing was defined as semiannual presentations of surveillance data to the participating hospitals and educational workshops held in each of these opportunities. Finally, in 2012, the Working Group in Healthcare-Associated Infections developed and issued a local technical note to guide healthcare institutions on the prevention and control of MDRO.^10^

### Participants

All hospitals with intensive care unit (ICU) that adhered to the notification program were included. Hospitals with exclusive maternity care were excluded.

### Variables and data source

All data were made available by the Municipal and State Health Departments, and to characterize the group of selected hospitals, data were collected regarding the number of beds and administrative area (private or public). Information related to the number of hospitalizations was also included. For HCAIs definitions, the use of the national criteria provided by the Brazilian Health Surveillance Agency ^11^, which is based on the Centers for Disease Control and Prevention criteria, was recommended. Moreover, for laboratories that provided microbiological support to hospitals, the use of Clinical and Laboratory Standards Institute for microbial sensitivity test parameters was suggested and about antimicrobial susceptibility methods, conventional (Kirby–Bauer) or any automated method of susceptibility test were accepted. The notification of pathogens as an etiological agent of HCAIs was performed in a grouped manner by the defined site of infection.

According to the Municipal Health Department Surveillance Program, the reported pathogens included subgroups of non-fermenting Gram-negative, fermenting Gram-negative, and Gram-positive bacteria and fungi. For each group of bacteria, specific antimicrobial susceptibility was monitored. However, based on the purpose of this study, only data on carbapenems and Gram-negative bacteria were analyzed. Hospitals were instructed to report susceptibility to carbapenems in *P. aeruginosa*, *A. baumannii*, *K. pneumoniae*, *E. coli*, *Enterobacter cloacae*, and other *Enterobacterales* that included *Serratia* spp., *Citrobacter* spp., *Proteus* spp., *Morganella* spp. in a grouped manner. A colonization report was not included in this analysis.

### Statistics analysis

Data on the number of admissions, number of HCAIs, and notified pathogens related to each site of infection were collected. The incidence rate of HCAIs was estimated using the number of HCAIs per 100 admissions. Carbapenem susceptibility was calculated using the number of carbapenem-susceptible (meropenem and/or imipenem) notified cases in the defined site divided by total number of that specific pathogen at the same site. The *Joinpoint Regression Program 4.6.0.0* was used to verify the trend related to carbapenem susceptibility in Gram-negative bacteria.^12^ The slope was significantly different from zero at the alpha level of 0.05 and 95% confidence interval (CI). Data were grouped in semiannual periods; therefore, the semiannual percent change (SAPC) provided by the program was related to 6-month periods. The software *IBM SPSS Statistics 23.0* was used for descriptive statistics on infection rates and susceptibility profile.

## Results

### Profile of included institutions

Out of the 33 hospitals with ICU in Curitiba, 29 (87.8%) participated in the monthly notification of microorganisms since the surveillance program started in 2010. Of these, five have an exclusive maternity profile and were thus excluded. Therefore, data from 24 hospitals were included in our analysis.

Regarding institutions characteristics, 13 are private, five are public, and six have a mixed profile (public and private). Of the total number of beds, 31% (1297) are in private hospitals, 29% (1222) in public hospitals, and 40% (1652) in those with mixture profile. Seventeen establishments have only adult care, six have both adult and pediatric care, and one hospital has only pediatric care.

### HCAI incidence rate

During the study period, 39,832 new HCAIs were noted in 1,503,634 admissions (2.65%; minimum 0.22%; maximum 12.41%).

### Microorganisms reported in HCAIs

The majority of notified microorganisms in HCAIs were Gram-negative (Table 1). Non-fermenting Gram-negative microorganisms were predominant in RTI. However, in UTI and SSI, fermenting Gram-negative microorganisms were the most frequent. In BSI, altogether fermenting and non-fermenting Gram-negative microorganisms outnumbered Gram-positive microorganisms.

**Table 1:** Distribution of microorganisms notified in HCAIs, in 24 hospitals with ICUs (2012–17).

HCAI site	BSI		RTI		UTI		SSI		Total	
Microorga-nisms	*N*	%	*N*	%	*N*	%	*N*	%	*N*	%
GN-NF	904	13.0	2,526	40.3	759	13.5	788	16.8	4,977	21.1
GN-F	2,514	36.0	2,484	39.7	4,106	72.9	2,144	45.6	11,248	47.7
GP	2,975	42.6	1,253	20.0	769	13.6	1,770	37.6	6,767	28.7
FG	586	8.4	a		a		a		586	2.5

Total	6,979	100.0	6,263	100.0	5,634	100.0	4,702	100.0	23,935	100.0

HCAIs, healthcare-associated infections; GN-NF, Gram-negative non-fermenter; GN-F, Gram-negative fermenter; GP, Gram-positive; FG, fungus; BSI, bloodstream infection; RTI, respiratory tract infection; UTI, urinary tract infection; SSI, surgical site infection; ICUs, intensive care units.

a Data not requested.

The frequency of Gram-negative species varied according to the HCAI site (Table 2). In SSI, *E. coli* (20.7%), *K. pneumoniae* (20.7%), and *Enterobacter* spp. (16.1%) were the most frequently notified Gram-negative pathogens. In RTI, the first ranked Gram-negative pathogens were *P. aeruginosa* (28.7%), *K. pneumoniae* (24.9%), and *A. baumannii* (21.7%). In BSI, *K. pneumoniae* was the most frequent Gram-negative pathogen (32.1%), followed by *Enterobacter* spp. (14.5%) and other *Enterobacterales* (14.5%). Finally, in UTI, *E. coli* was the most frequent pathogen (33.3%), followed by *K. pneumoniae* (30.8%) and *P. aeruginosa* (12.6%).

**Table 2:** Carbapenem susceptibility profile of Gram-negative HCAIs, in 24 hospitals with ICUs (2012–17).

Gram-negative	SSI		Carb-S		RTI		Carb-S		BSI		Carb-S		UTI		Carb-S	
	*N*	%	*N*	%	*N*	%	*N*	%	*N*	%	*N*	%	*N*	%	*N*	%
*E. coli*	608	20.7	594	97.7	261	5.2	259	99.2	424	12.4	409	96.5	1,621	33.3	1,603	98.9
*K. pneumoniae*	608	20.7	310	51.0	1,245	24.9	661	53.1	1,098	32.1	614	55.9	1,496	30.8	810	54.1
*Enterobacter* spp.	473	16.1	428	90.5	472	9.4	434	91.9	495	14.5	439	88.7	517	10.6	459	88.8
*Other Enterobacterales*	455	15.5	422	92.7	506	10.1	459	90.7	497	14.5	457	92.0	472	9.7	430	91.1
*P. aeruginosa*	497	17.0	418	84.1	1,437	28.7	932	64.9	463	13.5	315	68.0	611	12.6	423	69.2
*A. baumannii*	291	9.9	66	22.7	1,089	21.7	168	15.4	441	12.9	114	25.9	148	3.0	33	22.3

Total	2,932	100.0	2,238	76.3	5,010	100.0	2,913	58.1	3,418	100.0	2,348	68.7	4,865	100.0	3,758	77.2

HCAIs, healthcare-associated infections; BSI, bloodstream infection; RTI, respiratory tract infection; SSI, surgical site infection; UTI, urinary tract infection; Carb-S, carbapenem-susceptible; ICUs, intensive care units; *N*, number.

### Susceptibility profile

Regarding carbapenem susceptibility profile (Table 2), *E. coli* preserved susceptibility in all infection sites (>95%). In contrast, *A. baumannii* had the lowest susceptibility in all sites (15.4–25.9%). Carbapenem susceptibility of *K. pneumoniae* varied from 51% to 54%, considering the HCAI main sites. Furthermore, *P. aeruginosa* seemed to preserve carbapenem susceptibility in SSI (84.1%) but showed lower susceptibility (64.9–69.2%) in other sites.

Regarding trends in susceptibility profile (Table 3), some Gram-negative microorganisms showed changes in their trends. Notably, *K. pneumoniae* showed a 10.71% reduction in carbapenem susceptibility between 2012 and 2014, followed by a significant change in the tendency reflecting one *Joinpoint*, with an increase of 6.54% in the susceptibility profile from 2015 to 2017.

**Table 3:** Carbapenem susceptibility trend analysis in Gram-negative bacteria recovered in HCAIs (2012–17).

Gram-negative bacteria noted in HCAIs (all sites)	SAPC 2012–2017	*Joinpoint*	95% CI	*p*-Value
	Carb-S%			
*E. coli*	−0.02 (2012–2017)	0	−0.24, 0.19	0.82
*K. pneumoniae*	−10.71 (2012–2014)	1	−18.02, −2.75	0.02
	6.54 (2015–2017)		−2.00, 15.83	0.12
*Enterobacter* spp.	0.53 (2012–2017)	0	−0.24, 1.30	0.16
*Other Enterobacterales*	9.60 (2012–2013)	1	5.48, 13.88	0.00
	0.08 (2013–2017)		−0.23, 0.39	0.57
*P. aeruginosa*	1.49 (2012–2017)	0	−0.17, 3.17	0.07
*A. baumannii*	−2.49 (2012–2017)	0	−7.26, 2.52	0.29

HCAIs, healthcare-associated infections; SAPC, semiannual percent change by *Joinpoint* regression; CARB, carbapenem; CI, confidence interval; Carb-S, carbapenem-susceptible.

**Fig. 1:**
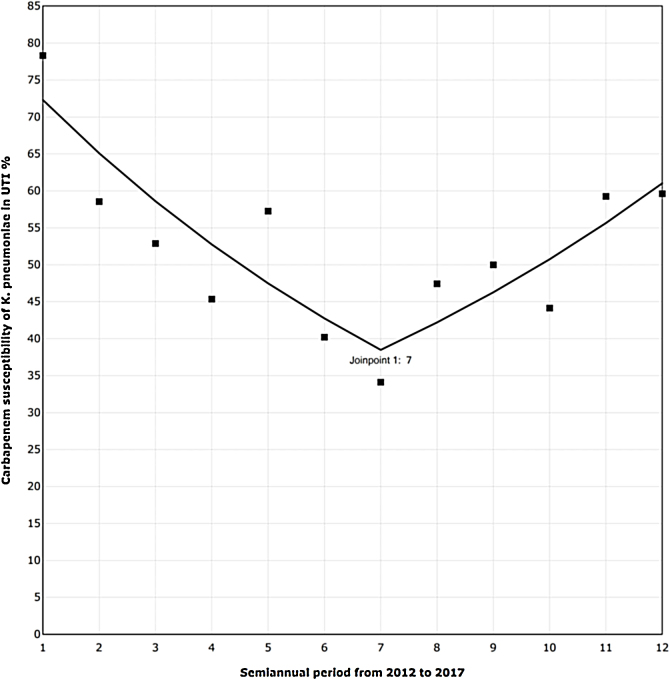
Carbapenem susceptibility trend analysis per semester in *Klebsiella pneumoniae* recovered in UTI, in 24 hospitals with ICUs (2012–17). ▪Observed SAPC (1–7): −9.98*; SAPC (7–12): 9.66. UTI, urinary tract infection; ICUs, intensive care units; SAPC, semiannual percent change by *Joinpoint* regression.

The trend analysis for *K. pneumoniae* in each infection site (Table 4) showed similar variation only in UTI (Fig. 1). First, from 2012 to the first semester of 2015, the SAPC (95% CI) was −9.98% (−16.03, −3.48; *p* = 0.01). Subsequently, the SAPC (95% CI) was 9.66% (−1.75, 22.39; *p* = 0.09).

**Table 4:** Carbapenem susceptibility trend analysis in *K. pneumoniae* noted in HCAIs per site (2012–17).

HCAI site	SAPC	*Joinpoint*	95% CI	*p*-Value
SSI	−3.93 (2012–2017)	0	−8.19; 0.52	0.08
RTI	−1.74 (2012–2017)	0	−5.48; 2.15	0.34
BSI	−2.02 (2012–2017)	0	−4.80; 0.83	0.14
ITU	−9.98 (2012–2014)	1	−16.03; −3.48	0.01
	9.66 (2015–2017)		−1.75; 22.39	0.09

SAPC, semiannual percent change by *Joinpoint* regression; CI, confidence interval; BSI, bloodstream infection; RTI, respiratory tract infection; UTI, urinary tract infection; SSI, surgical site infection; ICUs, intensive care units.

## Discussion

The most important findings in our study were as follows: (a) Gram-negative pathogens were the most frequent in all infection sites, and carbapenem susceptibility was lowest in *A. baumannii*, followed by *K. pneumoniae* and *P. aeruginosa.* (b) *K. pneumoniae* was the most common pathogen in BSIs with relevant carbapenem resistance in about half of the notifications. (c) The carbapenem susceptibility profile of *K. pneumoniae* has changed at a certain point in the study period, most influenced by UTI.

Regarding the proportion of Gram-negative pathogens in HCAIs, according to Allegranzi,^1^ Gram-negative bacilli were the most common nosocomial isolates, in both mixed populations and high risk-patients, based on information on pathogens causing HCAIs in developing countries in 28 of 220 selected studies.^1^ Moreover, among Gram-negative pathogens, carbapenem nonsusceptible (Carb-NS) *A. baumannii* is considered one of the most relevant pathogens at the WHO priority list of antibiotic-resistant bacteria.^4^

In fact, one of the major concerns on carbapenem resistance in our study is related to *A. baumannii.* Multidrug-resistant *A. baumannii* has been described in several outbreaks,^13^, ^14^ and clonal dissemination is greatly important in these events. For example, in a study conducted in eight hospitals of PR, Brazil,^15^ the molecular epidemiological features from 46 strains of Carb-NS *A. baumannii* in 2009–2011 showed that all isolates carried the *bla* Oxacilinase (OXA)-23 gene. Seven clones were identified, and the main cluster included 32 (69.5%) of the total isolates, even though hospitals were several kilometers apart. Thus, we believe that our data, in addition to the presented discussion, could alert authorities and institutions to develop strategies aiming at better control.

*P. aeruginosa* is also a common pathogen in HCAIs, especially in RTI. A 1-day point prevalence survey in 28 Brazilian ICUs identified that 40.5% of the 303 analyzed patients had at least one ICU-acquired infection.^16^ The most frequent infections included pneumonia (53.0%) and BSI (27.6%), and *P. aeruginosa* was the most common pathogen in pneumonia (30.4%). Our data showed similar findings with occurrence of 28.7% of 5010 notified bacteria in RTI.

According to the National Healthcare Safety Network report in 2015–2017, among 15,706 tested isolates of *P. aeruginosa* in device-associated infections 20.7% were resistant to carbapenems.^17^ Our data identified that out of 932 notified carbapenem resistant RTI, 35.1% of was caused by this pathogen, underscoring the relevant occurrence at this site of infection. Meanwhile, regarding the molecular epidemiology of this carbapenem resistance, one study conducted in Paraná (Brazil), between January 2009 and December 2012, identified that SPM-1 (São Paulo metallo-beta-lactamase) was the main metallo-beta-lactamase identified in Carb-NS *P. aeruginosa* in southern Brazil, with genetic similarity among some isolates suggesting clonal expansion.^18^

Shifting focus to carbapenem-resistant *Enterobacterales* (CRE), which includes *K. pneumoniae,* we presented in our study that this was the most common pathogen in BSIs with considerable carbapenem resistance. In a Brazilian nationwide surveillance study between 2007 and 2010 in 16 hospitals, Gram-negative organisms caused 58.5% of 2563 BSIs, in which *Klebsiella* spp. and *Acinetobacter* spp. were the most prevalent Gram-negative pathogens.^7^

In fact, it is interesting to mention that CRE has become increasingly prevalent worldwide, mainly by harboring mobile genetic elements.^19^ In Curitiba, PR (Brazil), the clonal diversity of several isolates from clinical samples and rectal swabs obtained between April 2010 and July 2012 was detected in 641 positive samples for *KPC*-2-producing *K. pneumoniae* (*Klebsiella pneumoniae* carbapenemase). Moreover, in 129 samples randomly selected for clonality evaluation, seven different clones were identified, showing the polyclonallity of the Curitiba outbreak.^20^

In addition to the potential for clonal dissemination, using invasive medical devices and carbapenem consumption are also important factors that can facilitate acquiring CRE in the setting of a local outbreak.^21^ Thus, efforts should be focused on the prevention and control of this dissemination, as it happened in Israel.^22^ In this country, there was no significant change in the proportion of Carb-NS *K. pneumoniae* isolates in BSI between 2014 and 2017 (4.6 to 4.0%, *p* = 0.36). According to these authors, the lowest proportion of Carb-NS *K. pneumoniae* observed in Israel occurred in the context of a national effort to control an outbreak witch expanded the actions of prevention, control, and resistance monitoring in the country.^23^

Regarding the susceptibility profile trend, our data analysis showed a reduction in carbapenem susceptibility in *K. pneumoniae* between 2012 and 2014, but from 2015, an improvement was observed, mostly influenced by UTI. It is not possible to affirm which reasons were related to this change, since this study did not include analysis of directed interventions by each institution. However, as it is an ecological study, it is possible to raise questions that could have influenced this finding.

Effective control strategies to prevent the establishment of CRE endemicity in a region include early detection through target laboratory protocols and containment of spread through comprehensive infection control measures.^3^ Furthermore, as the duration of urinary catheterization is the predominant risk factor for catheter-associated UTI, preventive measures directed at limiting the placement and early removal or urinary catheters can significantly reduce these infection rates.^24^ Finally, it is important to determine the influence of carbapenem consumption on CRE control. A study that analyzed data of 134 patients with CRE infection between 2011 and 2016, at a tertiary care hospital in Thailand, showed an increase in CRE incidence rate of 1.02 per 100,000 patient-days for a defined daily dose per 1000 patient-days increase in carbapenem consumption (*p* < 0.001).^25^ Our study did not include antimicrobial monitoring data as this information had not been requested by the municipal health department to hospitals until then. However, we believe that efforts should be made in this direction to incorporate this surveillance in the prevention and control measures of CRE.

Overall, we believe the results of our study provide support for infection control teams and stewardship efforts to improve and monitor infection control measures besides antibiotic consumption at local healthcare institutions along with bacterial resistance.

Some limitations of our study include the ecological nature and unavailability of laboratory confirmation by case of notified isolates in HCAIs, as data were reported by the hospitals in a grouped manner. Furthermore, as the surveillance program did not request separated notifications inside and outside the ICUs, it was not possible to detail findings more specifically related to ICU and device associated HCAIs. Also, as for this analysis, the data on patient-days per institution was not available, it was not possible to calculate the incidence density per 1000 patient-days for carbapenem-resistant Gram-negative bacteria. Finally, the possibility of underreporting or even some manual errors in HCAIs data notification by the hospital team should be considered, which could not be controlled in our study, generating residual bias.

In conclusion, the analysis presented in this study consists of important and updated surveillance information to support health institutions and authorities to develop control and prevention actions. Moreover, it should encourage future research on interventions capable of improving local epidemiology of microbial resistance, such as the establishment of directed protocols for antimicrobial treatment of infections, improvement in hospital infection control programs, and reduction of carbapenem consumption.

## Financial disclosures

None declared.

## Conflicts of interest

The authors declare no conflicts of interest.
